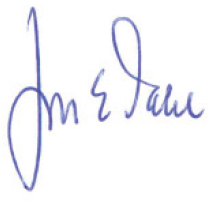# Recipients of Biomaterial Investigations in Dentistry’s Young Author Award 2024

**DOI:** 10.2340/biid.v12.44694

**Published:** 2025-09-01

**Authors:** Anne Peutzfeldt, Jon E. Dahl

**Figure:**
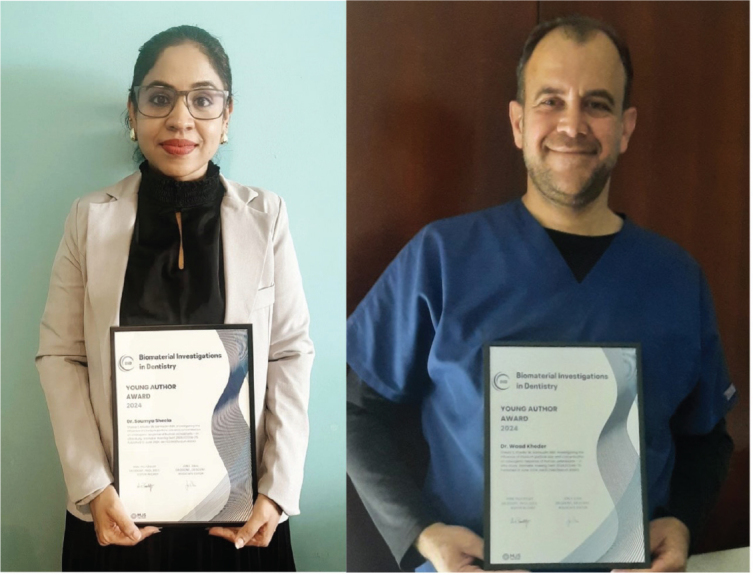


Dear Reader,

We are delighted to announce the recipients of the 2024 Young Author Award: Dr. Soumya Sheelaa and Dr. Waad Khederb (shared first authorship) from the Research Institute for Medical and Health Sciencesa and College of Dental Medicineb, University of Sharjah, United Arab Emirates. Drs. Sheela and Kheder receive the award for their paper:

“Investigating the influence of titanium particle size and concentration on osteogenic response of human osteoblasts – in vitro study” . The paper was published online on June 13, 2024, and co-authored by Dr. AB Rani Samsudin.

The nomination was motivated as follows: The paper reports on a comprehensive study, applying state-of-the-art, in vitro methods for biological evaluation. The results are presented in detail accompanied by figures and illustrating im-ages. Despite the lengthy discussion needed to thoroughly discuss the many results, the paper has a natural flow that allows the reader to focus on the acquired knowledge.

There were five eligible candidate papers, and the papers were evaluated based on the following criteria: originality of the study, suitability of the study design, presentation of the results, and readability of the paper.

The award is accompanied by a prize of € 5.000 Euro and a diploma.

The award aims to encourage young scientists to publish their research in Biomaterial Investigations in Dentistry and to showcase what is a good manuscript. The award is presented to a first author who at the time of submission of his/her manuscript is within 10 years of completing his/her last terminal degree (PhD, DDS, DMD, MD, etc.).

Our sincere congratulations to Dr. Sheela and Dr. Kheder and their colleague.


Anne Peutzfeldt Dr.Odont., Ph.D., D.D.S. *Editor-in-Chief* 
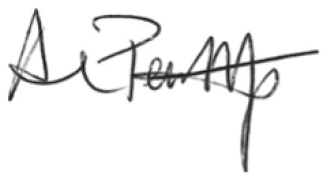

Jon E. Dahl Dr. Odont., Dr. Scient. *Associate Editor*